# Handgrip Strength of World Trade Center (WTC) Responders: The Role of Re-Experiencing Posttraumatic Stress Disorder (PTSD) Symptoms

**DOI:** 10.3390/ijerph16071128

**Published:** 2019-03-29

**Authors:** Soumyadeep Mukherjee, Sean Clouston, Roman Kotov, Evelyn Bromet, Benjamin Luft

**Affiliations:** 1 Community Health and Wellness, Health & Physical Education Department, Rhode Island College, Providence, RI 02908, USA; smukherjee@ric.edu; 2 Program in Public Health, Department of Family, Population, and Preventive Medicine, Renaissance School of Medicine at Stony Brook University, Stony Brook, NY 11794, USA; 3 Department of Psychiatry, Renaissance School of Medicine at Stony Brook University, Stony Brook, NY 11794, USA; roman.kotov@stonybrookmedicine.edu (R.K.); evelyn.bromet@stonybrookmedicine.edu (E.B.); 4 World Trade Center Health and Wellness Program Director, Department of Medicine, Renaissance School of Medicine at Stony Brook University, Stony Brook, NY 11794, USA; Benjamin.Luft@stonybrookmedicine.edu

**Keywords:** 9/11 disaster, handgrip strength, WTC responders, PTSD, depression, aging

## Abstract

*Background*: This study sought to examine whether handgrip strength (HGS), a measure of muscle strength and a biomarker of aging, was associated with post-traumatic stress disorder (PTSD) in a cohort of World Trade Center (WTC) responders at midlife. *Methods*: HGS was assessed utilizing a computer-assisted hand dynamometer administered to a consecutive sample of men and women (*n* = 2016) who participated in rescue and recovery efforts following the World Trade Center (WTC) attacks and subsequently attended monitoring appointments in Long Island, NY. PTSD symptom severity and depressive symptoms were assessed using the PTSD specific-trauma checklist (PCL-S) and the Patient Health Questionnaire (PHQ-9). General linear models were used to examine the association of WTC-related PTSD with HGS after adjusting for confounders. *Results*: The sample was at midlife (mean age = 53.3) when assessed, and 91.3% were men. Nearly 10% of the sample had probable PTSD (PCL ≥ 44) with concomitant depression (PHQ ≥ 10), while 5.1% had probable PTSD without depression. Average HGS was 57.4 lbs. (95% confidence interval (95% CI): 56.6–58.1) among men and 36.1 lbs. (95% CI = 33.8–38.5) among women. Mean HGS of those with probable PTSD with concomitant depression was lower (45.9 lbs., 95% CI = 43.6–48.2) than responders with only PTSD (49.1 lbs., 95% CI = 46.0–52.4) and those without PTSD or depression (57.5 lbs., 95% CI = 56.2–57.8). Subdomain analyses of PTSD symptoms revealed that re-experiencing symptoms at enrollment (*p* = 0.003) was associated with lower HGS after adjusting for depressive symptoms and other confounders. *Discussion*: Results suggested that higher WTC-related PTSD symptom severity was associated with lower HGS. Results support ongoing work suggesting that PTSD may be associated with more rapid physical aging. The potential for developing interventions that might simultaneously improve physical and mental health in the aftermath of trauma may be considered.

## 1. Introduction

Hand grip strength (HGS) is an indicator of upper body strength that quantifies the amount of static force with which a person’s hand can squeeze a dynamometer [1]. Often used as a marker of overall muscular strength, HGS is negatively associated with physical frailty [2]. Diminishing HGS is associated with muscle wasting and is commonly observed in older age, suggesting that it may act as a biomarker for aging. Poorer HGS is also associated with increased disability and mortality [3].

In addition to physical health, HGS has shown association with mental health outcomes. For example, a number of studies examining adults in midlife and older have reported that depressive symptoms were associated with HGS [4,5], while another study among Australians aged 85 years and older also found that lower HGS was associated with worse functional, psychological, and social health [3]. Additionally, higher HGS was shown to be associated with better cognitive performance among older participants with major depression, and those with bipolar disorder, as well as among healthy controls [6] and, in a second study maximal HGS was found to be associated with cognitive performance in the general population, and among those with schizophrenia [7].

Despite associations with a variety of health outcomes, to date no work has examined associations between HGS and symptoms of posttraumatic stress disorder (PTSD) in individuals who have experienced a severe trauma. Our recent work has suggested that PTSD may increase risk of cognitive aging [8] and physical functional limitations [9], suggestive of the potential for more rapid aging. This study therefore sought to determine whether weaker HGS is also associated with PTSD. Since PTSD is often comorbid with depression, we further examined whether the association, if any, of HGS with PTSD was magnified by the presence of depression. Lastly, since PTSD is a heterogeneous disorder, we further examined whether HGS had differential associations with PTSD symptom domains.

## 2. Materials and Methods

### 2.1. Participants and Procedure

The Centers for Disease Control and Prevention, in the year 2002, established a monitoring and treatment program for those who responded to the World Trade Center (WTC) terrorist attacks of 11 September 2001 by participating in rescue and recovery efforts. Since then, more than 33,000 responders have enrolled in the WTC general responder population (GRP) [10]. The WTC Wellness Program at Stony Brook University (SBU) monitors more than 8000 of these responders primarily residing on Long Island, NY. This SBU cohort is similar in terms of exposure, sex, and age to those enrolled in the GRP [10], with the majority being males (92.0%), most of them working in law enforcement (71.5%) during 9/11, with an average age of 38.9 years at the time of the WTC attack. Most SBU responders (85.0%) have continued to return to either Nassau or Suffolk county clinics within 18 months of their prior visit.

In 2016, the SBU WTC Wellness program began assessing responders’ HGS as a part of their functional assessment. The current study includes a consecutive sample of responders (*n* = 2016) who were monitored between April 2016 and September 2017. The study was approved by the Stony Brook University Ethics Review Board (IRB approval No. 604113); responders provided informed, written consent. In comparison with the SBU population of responders who were eligible for research but did not complete the functional assessment, those who completed the functional assessment were 0.85 years younger (*p* < 0.001) and were less likely to be women (*p* = 0.004), but had similar occupations on 11 September 2001 (*p* = 0.115), showed similar levels of current PTSD (*p* = 0.521), and similar baseline PTSD Checklist (PCL) scores (*p* = 0.455).

### 2.2. Assessment of HGS

HGS was assessed for both hands only if responders did not self-report any trouble with their hands, shoulders, or wrists and if they felt that they would be able to safely complete the assessment. Vernier dynamometers were administered by trained healthcare providers to measure and record HGS among responders sitting upright in chairs with arm rests such that their elbow formed close to a right angle while they squeezed the dynamometer between their thumb and other fingers with maximum strength for 10 s. Each responder completed two trials for each hand beginning with the left hand. Force was measured in pounds (lbs.) and recorded in a computer with Logger Pro software (Vernier® Software & Technology, Beaverton, OR, USA). The data were exported as. csv files, cleaned and the maximum HGS was used for this study. The average of two trials were computed, followed by the average of two hands which was the outcome for this study.

### 2.3. Assessment of PTSD Symptoms

PTSD symptoms were assessed at each monitoring visit using the PTSD checklist, specific trauma version tailored to the WTC disaster (PCL-S trauma specific version) [11]. At each monitoring visit, respondents rated the extent to which they were bothered by 17 DSM-IV WTC-related PTSD symptoms during the past month on a scale from 1 (not at all) to 5 (extremely). The PCL has good internal consistency and convergent validity [12]. The total PCL score was used to categorize respondents into those who had or did not have probable WTC-related PTSD, using a cut-off of 44 [11]. Four PTSD symptom clusters were also analyzed: avoidance, hyperarousal, negative affect and re-experiencing symptoms.

### 2.4. Assessment of Depressive Symptoms

Symptoms of depression were measured by the Patient Health Questionnaire with nine items (PHQ-9). Probable depression was indicated by a PHQ-9 score of 10 or higher. WTC responders were categorized based on whether they had both probable PTSD and probable depression; probable PTSD without depression; or neither PTSD nor depression. Depression only group was not considered because of very few responders who had depression without PTSD symptoms.

### 2.5. Covariates

A number of variables that could be potentially associated with health and functional status were considered including age in years at the time of HGS assessment and gender. According to educational attainment, responders were categorized into those who did not finish high school, those who graduated high school, those with some college education, those with an undergraduate degree, and those who have been to graduate school. Race/ethnicity was categorized as non-Hispanic White, non-Hispanic Black, Hispanic, Asian, and others. Responders were categorized as single, married, and separated or divorced or widowed depending on their marital status. Current employment status was given as working full time, working part time, being disabled or on medical leave, retired, and laid off or unemployed. Annual household income (US$) was categorized as 50,000 or less, more than 50,000 to 70,000, more than 70,000 to 100,000 and higher than 100,000. According to their self-reported general health, responders had excellent, very good, good, fair, or poor general health. Responders’ self-reported hand-dominance was taken into account to classify them as right-handed, left-handed, or ambidextrous.

Taking into account the severity of responders’ exposure to the WTC disaster, four exposure groups—very high, high, intermediate, and low—were created based on the work of Wisnivesky et al. [13]. The very high exposure group comprised of those who worked more than 90 days at the WTC site, were exposed to the dust cloud due to the collapse of WTC buildings, and worked at least some time on the pile of debris. Rescue workers who were exposed to the dust cloud but either worked on-site less than 90 days or did not work on the debris pile, were categorized to have had high exposure. Workers with intermediate exposure were exposed to the dust cloud and either worked between 40 days and 90 days or did not work on the debris pile. Workers in the lowest exposure group worked less than 40 days on-site, were not exposed to dust from the collapse, and did not work on the debris pile.

### 2.6. Statistical Analysis

Descriptive sample statistics, including means and standard deviations of maximum HGS were computed. Average of two runs from each hand was calculated followed by the average from both hands. Means and/or proportions were computed for all independent variables and covariates.

Linear regression was performed to examine bivariate associations of mean HGS with WTC-related probable PTSD and all other independent variables. Mean HGS were compared between different levels of each variable and 95% confidence intervals (CI) were computed for the means. Estimates of regression coefficients and their respective p-values were reported. A two-tailed *α* = 0.05 was utilized to determine statistical significance. Multivariable general linear models were used to examine the association of WTC-related PTSD with HGS after adjusting for covariates.

In separate analyses, scores for individual PTSD domains were used to examine whether re-experiencing symptoms, avoiding situations reminiscent of the WTC event, negative changes in beliefs and feelings, or hyperarousal symptoms were associated with HGS, after adjusting for depression (PHQ-9) scores and other covariates.

## 3. Results

The mean age of the sample was 53.3 (SD: 7.9) years with a range from 35.1 to 85.0 at the time of their HGS assessment, and 91.3% were men (Table 1). 

The majority (86%) were non-Hispanic White (NHW). The average maximal HGS was 55.5 lbs. (SD 17.1) with a minimum of 7.5 lbs. and a maximum of 109.0 lbs. There was no significant difference in HGS and hand dominance. There was a decrease in maximum HGS with increasing age: every year older age was associated with a decrease in maximum HGS by 0.5 lbs. (*p* < 0.0001) (Table 2). Women had mean maximum HGS being more than 21 lbs. lower than men. Among racial/ethnic groups, non-Hispanic Blacks had significantly lower HGS than NHWs. Those who were on disability or on medical leave and those who were retired at the time of the study had significantly lower HGS than those who were working full-time on 9/11/2001. Having fair or poor self-reported general health was associated with lower HGS than those with excellent health. Mean HGS of responders with probable PTSD with concurrent depression was 11 lbs. lower (*p* < 0.0001), whereas mean HGS of responders with probable PTSD without depression was 8 lbs. lower (*p* < 0.0001) than those without either PTSD or depression.

In multivariate analysis, being in fair or poor health was associated with lower HGS than those with excellent health (Table 2). Responders with both WTC-related probable PTSD and depression, but not those with PTSD without depression, had significantly (*p* = 0.0005) lower mean HGS than those without PTSD or depression. Higher age and female gender remained significantly associated with lower HGS, while race/ethnicity was no longer statistically significant. Mean HGS of those who were disabled or on medical leave and those retired was significantly lower than those working full-time. While income levels had no significant relationship in bivariate analyses, multivariable analysis revealed that HGS of responders with income between US$ 70,000 and 100,000, and those with annual income higher than US$ 100,000 were higher by 3lbs. (*p* = 0.04) and 4 lbs. (*p* = 0.01) respectively, in comparison with those earning US$ 50,000 or less. Levels of WTC exposure did not show significant relationship with mean HGS in either bivariate or multivariate analyses.

### Subdomain Analyses

On further examination of the PTSD domains (results not shown in table), higher severity of re-experiencing symptoms was significantly associated with lower HGS while all other domains showed no such relationship after controlling for other domains and all other covariates. Increase in re-experiencing score by one point was associated with a decrease in HGS by 0.45 lbs. (*p* = 0.0032); since those with the worst symptom severity have symptom scores 15 or 20 points above those with no symptoms, this translates into substantial and large differences in HGS. 

## 4. Discussion

Lower HGS, after adjusting for sex, is increasingly seen as a biomarker of aging [5,14,15,16,17]. Our study similarly revealed that max HGS was associated with older age, supporting this view. Additionally, our study revealed that those with probable WTC-PTSD, with and without concomitant depression had significantly lower maximal HGS than those without either PTSD or depression. Examining individual PCL domain scores revealed that the re-experiencing symptom cluster was specifically associated with HGS, a finding that remained significant even after adjusting for covariates and other PTSD symptoms.

A remarkable finding was that the mean HGS of WTC responders in this study, many of whom were in the law enforcement and are usually shown to have “healthy worker” effects, is not as high as might typically be expected. Based on normative data published by Mathiowetz et al., the mean HGS of adults in the 50–54 year-old age range—the mean age of our sample—was 102–114 lbs. for men and 57–66 lbs. for women [18]. Among respondents to the *Health and Retirement Survey* in 2006–2012, the mean HGS of 55–59-year-old men was 38–43 kg or 84–95 lbs., while it was 22–28 kg or 49–62 lbs. for women [17]. By comparison, the mean HGS was 57.4 lbs. for men and 36.1 lbs. for women in our sample. Unlike our sample, which comprised of responders with varying degrees of exposure to the WTC trauma, both of the aforementioned studies involved civilian populations. Moreover, adjustable-handle Jamar® dynamometer and Smedley® spring-type dynamometer were used as opposed to the Vernier dynamometer used in our study. Future research needs to examine whether lower HGS in our cohort can be attributed to their exposure to the traumatic WTC experiences, and/or due to differences in the instruments used. Our findings should be interpreted with caution, however, as lower HGS among WTC responders may also be attributed to PTSD-related weakness, easy fatigability, and neurasthenia, as evidenced from symptoms experienced by war veterans [19].

Our finding of lower maximal HGS among WTC responders with WTC-related PTSD and depression are along the lines of those reported by Clouston et al. when current PTSD was associated with a two-fold higher risk of functional limitations as indicated by Short Physical Performance Battery (SPPB) scores of 9 or lower [9]. Specifically, current PTSD showed strong adjusted associations with slower walking speeds (<0.8 m/s), slower chair-rise speed (<0.39 rises/s), and balance problems. Previous research by Keller-Ross and colleagues (2014) found that veterans with PTSD have greater fatigability as well as greater fluctuations in force exerted by their handgrip muscles [20]. It is possible that PTSD affects physical functioning and HGS through similar pathways. Furthermore, depending on their mental health and motivation, not all responders may have exerted adequate effort when asked to take the HGS test. However, this concern may have been partially addressed as our analyses took depressive symptoms, in addition to symptoms of PTSD, into consideration. It is notable that of all the PTSD domains, re-experiencing symptoms emerged as most significantly associated with lower HGS. This is comparable to prior work where re-experiencing symptoms were found to be consistently associated with cognitive impairment among WTC responders [8]. Re-experiencing symptoms that include sudden intrusive memories of the traumatic event, nightmares, flashbacks, and other feelings of distress, have been noted to be early markers of mental pathology [21,22]. Findings from our current study suggest that these symptoms can affect individuals both physically and mentally. 

Future research may focus on the association between HGS and PTSD symptoms among populations with other traumatic experiences. There is some evidence that resistance training can lead to better cognitive functioning among the elderly through a posited reversal of age-related deterioration of the brain white matter [23,24]. It will be important to complete the clinical picture by examining other trauma-related symptoms, including depression. It might be useful to explore whether HGS improvement may help address some PTSD symptoms, in addition to the existing modes of management. It is also possible that changes in HGS precede changes in PTSD symptoms or higher HGS at baseline protects against psychological symptoms, as similar hypotheses have been postulated for cognitive decline as well. The integrity of white matter has been linked to optimum physical and mental functioning; therefore, the same underlying mechanisms could predispose responders to lower HGS as well as PTSD and depression symptoms.

### Limitations

This is the first study to examine HGS in a large sample of WTC responders, and the first to report associations between PTSD and HGS. A key limitation of this study is its cross-sectional nature, limiting our ability to investigate possible reverse causation. Since we found associations between PTSD symptoms measured at program enrollment visits, which occurred on average 8–10 years before HGS was assessed, the potential for reverse causation is greatly reduced. However, the relationship could be bidirectional, and a potential common pathology that predisposes to both PTSD symptoms and muscular weakness might be the most valuable take-away from our study. It is, therefore, critical that future work examine associations between PTSD symptoms and changes to HGS over time. The generalizability of this study to the general population, given that responders in this study are residents of Long Island, NY, with a majority being highly educated White males. The WTC responders were exposed to a very unique event—the 9/11 attacks and its aftermath—which may not result in similar physical exposures and, therefore, concomitant PTSD complexity, severity, or chronicity that is similar to symptoms experienced after other traumatic exposures. This study does not, as well, consider the impact of childhood exposures to stressful events though such events have been found to worsen mental health [25]. Nevertheless, many of the findings among veterans and healthy adults from different countries provide similar overall conclusions.

## 5. Conclusions

This, to our knowledge, is the first study examining the association of HGS with PTSD with or without coexisting depressive symptoms in a large cohort exposed to a severe traumatic event. Our finding that PTSD symptoms in general, and re-experiencing in particular, are associated with lower HGS fifteen years after a significant traumatic event could have clinical implications in the potential for HGS to be a “biomarker” of aging in the context of severe and chronic PTSD. Furthermore, and similar to prior findings, this points out to the potential for interventions targeting physical strength also having a beneficial effect on responders’ mental health, or vice versa.

## Figures and Tables

**Table 1:** Distribution of handgrip strength (HGS) with independent variables among World Trade Center (WTC) responders (*n* = 2023).

Characteristics	Mean (SD)/Number (%)	Mean HGS (lbs.) (95% CI)
Age in years	53.3 (7.9)	55.5 (54.8–56.3)
Gender		
Men	1847 (91.3)	57.4 (56.6–58.1)
Women	176 (8.7)	36.1 (33.8–38.5)
Race/ethnicity		
Non-Hispanic White 0	1613 (85.8)	56.2 (55.3–57.0)
Non-Hispanic Black 1	86 (4.6)	50.8 (47.2–54.4)
Hispanic 2	156 (8.3)	52.7 (50.0–55.4)
Asian 3	19 (1.0)	52.6 (44.9–60.3)
Other 4	7 (0.4)	47.9 (35.3–60.5)
Hand dominance		
Right	1721 (85.1)	55.4 (54.6–56.2)
Left	208 (10.3)	55.5 (53.1–57.8)
Ambidextrous	94 (4.7)	58.4 (54.9–61.8)
Marital status		
Single	107 (5.3)	49.9 (46.6–53.1)
Married	1678 (83.0)	56.1 (55.3–56.9)
Separated/Divorced/Widowed	238 (11.8)	53.8 (51.6–56.0)
Education		
Did not finish high school	55 (2.8)	51.6 (47.0–56.1)
Graduated high school	349 (17.8)	53.5 (51.7–55.3)
Some college	935 (47.7)	55.6 (54.5–56.7)
Undergraduate degree	467 (23.8)	57.6 (56.1–59.2)
Graduate school	155 (7.9)	55.0 (52.3–57.7)
Current employment status		
Working full time	1153 (57.6)	58.8 (57.8–59.7)
Working part time	147 (7.3)	56.9 (54.2–59.6)
Disabled/on medical leave	106 (5.3)	50.6 (47.5–53.8)
Retired	572 (28.6)	50.1 (48.7–51.4)
Laid off/unemployed	25 (1.3)	51.3 (44.8–57.8)
Annual income (USD)		
0 to 50,000	181 (11.7)	54.9 (52.5–57.4)
>50,000 to 70,000	428 (27.7)	55.2 (53.6–56.8)
>70,000 to 100,000	678 (43.9)	56.1 (54.8–57.4)
>100,000	258 (16.7)	56.4 (54.4–58.5)
Level of WTC exposure		
Low	331 (18.0)	53.9 (52.1–55.8)
Intermediate	1092 (59.4)	56.1 (55.1–57.1)
High	339 (18.5)	56.2 (54.4–58.0)
Very high	75 (4.1)	53.6 (49.8–57.5)
WTC-related PTSD		
Probable PTSD (PCL-S ≥ 44) with depression (PHQ-9 ≥ 10)	200 (9.9)	45.9 (43.6–48.2)
Probable PTSD without depression	104 (5.1)	49.2 (46.0–52.4)
No PTSD or depression	1719 (85.0)	57.0 (56.2–57.8)
Self-reported general health		
Excellent	87 (4.4)	61.4 (57.9–64.9)
Very good	461 (23.1)	59.6 (58.1–61.1)
Good	990 (49.7)	56.7 (55.7–57.7)
Fair	381 (19.1)	49.3 (47.6–50.9)
Poor	75 (3.8)	41.7 (38.0–45.5)

Total numbers vary between variables because only responders with valid response for each variable are included.

**Table 2:** Relationship of handgrip strength (HGS) with independent variables among WTC responders.

Characteristics	Bivariate Regression Coefficient (*p* Value)	Multivariable Regression Coefficient (*p* Value)
Age in years	−0.53 (<0.0001)	−0.52 (<0.0001)
Gender		
Men	Ref.	Ref.
Women	−21.23 (<0.0001)	−21.8 (<0.0001)
Race/ethnicity		
Non-Hispanic White	Ref.	Ref.
Non-Hispanic Black	−5.38 (0.04)	0.48 (0.81)
Hispanic	−3.44 (0.11)	−2.23 (0.15)
Asian	−3.58 (0.89)	−2.28 (0.53)
Other	−8.26 (0.70)	−15.74 (0.27)
Hand dominance		
Right	Ref.	Ref.
Left	0.09 (0.36)	−0.71 (0.54)
Ambidextrous	3.00 (0.22)	3.82 (0.05)
Marital status		
Single	Ref.	Ref.
Married	6.25 (0.0007)	0.91 (0.65)
Separated/Divorced/Widowed	3.94 (0.12)	1.46 (0.52)
Education		
Did not finish high school	Ref.	Ref.
Graduated high school	1.96 (0.93)	4.90 (0.06)
Some college	4.06 (0.42)	4.42 (0.08)
Undergraduate degree	6.07 (0.09)	4.59 (0.08)
Graduate school	3.47 (0.69)	3.01 (0.30)
Current employment status		
Working full time	Ref.	Ref.
Working part time	−1.84 (0.71)	−1.05 (0.51)
Disabled/on medical leave	−8.13 (<0.0001)	−5.04 (0.02)
Retired	−8.72 (<0.0001)	−2.17 (0.03)
Laid off/unemployed	−7.47 (0.17)	−6.41 (0.14)
Annual income (USD)		
0 to 50,000	Ref.	Ref.
>50,000 to 70,000	0.25 (0.99)	2.55 (0.08)
>70,000 to 100,000	1.18 (0.84)	2.90 (0.04)
>100,000	1.54 (0.78)	4.17 (0.01)
Level of WTC exposure		
Low	Ref.	Ref.
Intermediate	2.17 (0.18)	1.60 (0.15)
High	2.28 (0.31)	2.44 (0.07)
Very high	0.30 (0.99)	−3.93 (0.08)
WTC-related PTSD		
Probable PTSD (PCL-S ≥ 44) with depression (PHQ-9 ≥ 10)	−11.11 (<0.0001)	−5.33 (0.0005)
Probable PTSD without depression	−7.8 (<0.0001)	−3.04 (0.14)
No PTSD or depression	Ref.	Ref.
Self-reported general health		
Excellent	Ref.	Ref.
Very good	−1.76 (0.89)	−0.86 (0.69)
Good	−4.72 (0.08)	−1.72 (0.41)
Fair	−12.11 (<0.0001)	−7.27 (0.001)
Poor	−19.65 (<0.0001)	−15.52 (<0.0001)

Only responders with non-missing responses for each variable are included. Ref.: Reference category.
